# Web-Based Health Information Seeking by People Living With Multiple Sclerosis: Qualitative Investigation of the Multiple Sclerosis Online Course

**DOI:** 10.2196/53372

**Published:** 2024-02-09

**Authors:** William Bevens, Rebekah Davenport, Sandra Neate, Maggie Yu, Pia Jelinek, George Alexander Jelinek, Jeanette Reece

**Affiliations:** 1 Centre for Epidemiology and Biostatistics Melbourne School of Population and Global Health The University of Melbourne Carlton Australia; 2 Institute for Clinical and Translational Science University of California, Irvine Irvine, CA United States; 3 Melbourne School of Psychological Sciences The University of Melbourne Parkville Australia

**Keywords:** information-seeking behavior, self-management, lifestyle, digital health

## Abstract

**Background:**

Digital technologies have afforded people living with multiple sclerosis (MS) access to telehealth consultations, diagnostic tools, and monitoring. Although health care professionals remain the most trusted source of information, the internet has emerged as a valuable resource for providing MS-related information, particularly during the COVID-19 pandemic. Notably, people living with MS are increasingly seeking educational content for a range of topics related to the self-management of MS; however, web-based information seeking remains largely underevaluated. To address this gap and ensure that web-based health-related information is accessible and engaging, this study used qualitative methods to analyze the reflections from participants of web-based educational programs for people living with MS.

**Objective:**

This study aimed to explore the motivations, behaviors, and expectations of web-based health information seeking for people living with MS.

**Methods:**

We conducted semistructured interviews for 38 people living with MS 1 month after they completed the novel MS Online Course, which provided information on modifiable lifestyle-related risk factors for people living with MS. Of the 38 participants, 22 (58%) completed the intervention course and 16 (42%) completed the standard care course. Inductive thematic analysis was used within a qualitative paradigm, and 2 authors coded each interview separately and arrived at themes with consensus.

**Results:**

We identified 2 themes: motivation to learn and MS information on the web. The diagnosis of MS was described as a pivotal moment for precipitating web-based information seeking. People living with MS sought lifestyle-related information to facilitate self-management and increase control of their MS. Although social media sites and MS websites were considered useful for providing both support and information, discretion was needed to critically appraise information. Recognizable institutions were frequently accessed because of their trustworthiness.

**Conclusions:**

This study provided novel insights into the motivations of people living with MS for seeking web-based health information. Furthermore, their preferences for the content and format of the web-based information accessed and their experiences and reactions to this information were explored. These findings may guide educators, researchers, and clinicians involved in MS care to optimize the engagement and processing of web-based health information seeking by people living with MS.

## Introduction

### Background

Multiple sclerosis (MS) is a chronic neurodegenerative disease that affects almost 3 million people worldwide [1]. Despite the advent of a range of disease-modifying medications that can modestly affect disease progression, MS is associated with a large disease burden because of relatively early onset and the accumulation of disability with no cure available. Self-management approaches, in which individuals are encouraged to become active participants in the management of their disease [2,3], are highly relevant to people living with MS. MS-related knowledge and self-management skills are integral in assisting people living with MS to manage their disease [4]. Knowledge of and self-management skills for MS are mediated by how people living with MS access and engage with relevant health information sources [5]; however, this remains an underexplored area of MS care.

It is well understood that people living with MS seek information related to MS very early on in the disease course, often occurring before their first neurology appointment [6]. Much of this information is sourced on the web [6-8], whereby people living with MS report not only empowerment but also skepticism toward information found on websites [9,10] and social media [11]. Neurologists and other health care professionals (HCPs) can assist in critically appraising sourced information [12]; however, people living with MS often seek information independently of formal health care environments [13]. In the context of an increasingly complex digital world, structured educational programs from trusted providers may provide important mechanisms for health information delivery for people living with MS. Several groups have developed web-based programs to deliver educational content to people living with MS, with the goal of increasing MS-related knowledge [14,15], and have demonstrated early success [16]. What remains unexplored is how the self-directed web-based health information–seeking behavior of people living with MS relates to their engagement with educational intervention programs delivered via web-based platforms.

This qualitative study formed a part of a large randomized controlled trial (RCT) comparing the effects of 2 web-based educational lifestyle programs on medium- and long-term health outcomes in people living with MS. This study presents a qualitative analysis of semistructured interviews of participants conducted 1 month after the completion of the MS Online Course (MSOC) RCT [17].

### Objectives

This qualitative analysis specifically aimed to investigate health information seeking in a subset of people living with MS interested in lifestyle behavior modification (ie, those who selected to actively engage in a lifestyle education program via enrollment in the MSOC RCT). Notably, this study also sought to explore the perceptions of people living with MS encountering health information on the web and how they navigate different types of health information across various sources.

## Methods

### Ethical Considerations

This ancillary study was approved by the University of Melbourne Human Research Ethics subcommittee on November 2, 2021 (ID: 1851781.2). This protocol for the RCT was prospectively registered on November 25, 2021, and was approved by the Australian and New Zealand Clinical Trials Registry (ACTRN: ACTRN12621001605886). Written informed consent was obtained from all participants before inclusion in the RCT, with additional verbal consent obtained from all participants in this study.

### MSOC RCT Study Design

The MSOC RCT study design and course content have been comprehensively described previously [17]. We developed 2 courses in conjunction with people living with MS: the standard care course (SCC) based on the information from MS websites and the intervention course (IC) based on the Overcoming MS program. The content and format of the Overcoming MS program have been described comprehensively elsewhere [18]; however, briefly, it is a lifestyle management program recommending a plant-based wholefood diet, exercise and stress reduction activities, sunlight exposure, and vitamin D consumption. The 7 modules of the first round of the MSOC were delivered across 6 weeks from August to September 2022.

We recruited participants for the MSOC RCT web-based across many Facebook (Meta Platforms, Inc) groups and MS websites and forums worldwide, as previously described [17]. The consenting participants completed a screening questionnaire on the study website to confirm eligibility: a self-reported diagnosis of MS by a physician and aged at least 18 years. A baseline survey consisting of questions on sociodemographic information, lifestyle habits, and health status was included in the first module of the course. This baseline survey was voluntary for participants to complete, and consequently, only the participants who completed this baseline survey were included in the MSOC RCT for subsequent data analysis.

### Qualitative Recruitment

We collected qualitative data 1 month after course completion. The possibility of an invitation to participate in an interview in the poststudy period was outlined in the RCT consent form and plain language statement. All participants who completed the first round of the MSOC and baseline and postcourse evaluation surveys were emailed an invitation to participate in a semistructured interview along with a plain language statement and consent form specific for the qualitative interviews that outlined the time commitment and process.

### The Interviewers

The interviews were conducted by SN, JR, RD, and PJ, all with clinical and qualitative research expertise. We had access to the course materials and were not blinded to participants’ course allocation, which is a necessary step in understanding participants’ views on the course content. Single blinding of the participants was maintained.

### The Interviews

The question guide for the 1-month postcourse interview was developed by WB, RD, SN, PJ, and JR (provided in Multimedia Appendix 1 [19-23]).

We conducted interviews by videoconference using the Zoom (Zoom Video Communications, Inc) software licensed by the University of Melbourne or via WhatsApp. The first 3 interviews were conducted with a lead interviewer and 2 observers, with the permission of the interviewee, to assess the interview schedule and facilitate consistency between the interviewers. Following the first 3 interviews, the interview schedule was minimally revised, based on our observations, to reduce repetition and improve the clarity of the questions. Further interviews were conducted with 1 or 2 interviewers depending on our availability. The interviews were digitally recorded and transcribed verbatim using the voice recognition software [24].

The interviewer manually edited the transcripts while listening to the recordings to ensure accuracy. Edits to transcripts, including the removal of data, were made only for the following reasons:

1. Grammatical or spelling errors produced by the software during transcription
2. Removal of personal, sensitive, or other information that was deemed irrelevant to the overarching aims of the study

Transcripts were imported into NVivo (Lumivero) software for data management. In accordance with best practice data management, NVivo document coding development and theme evolution were maintained within the NVivo outputs [25].

### Qualitative Data Analysis

**Figure 1:**
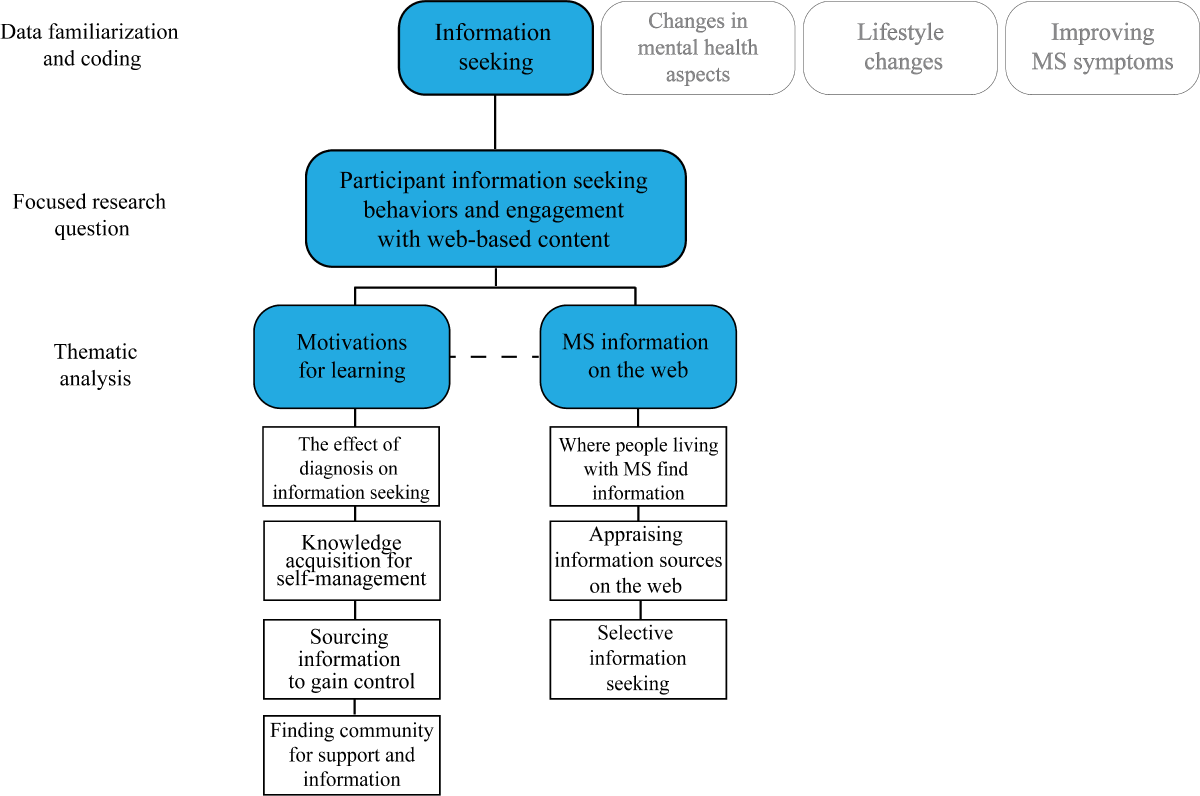
Process of the development of codes, themes, and subthemes. MS: multiple sclerosis.

The primary analytical approach to this data set was an inductive, reflexive thematic analysis (RTA), which allows for epistemological flexibility when determining theories from which to interpret data [26]. The strengths of RTA allow for researcher autonomy on what type of theme to identify (eg, latent or semantic) and what type of analysis to apply (inductive vs theoretical) [27]. Despite its flexibility, RTA still requires a systematic approach to analysis, which is categorized into 6 distinct phases [26]. WB and RD performed data immersion by reviewing all the transcripts and checking against the recordings to ensure transcript accuracy. Initial code generation was then completed separately using the NVivo software by WB and RD. In phase 3, WB and RD initially divided the data corpus between the IC and SCC arms and independently generated codes and themes from 1 arm at a time. The analysis team (WB, RD, SN, and JR) met to examine these codes, and it was determined at this stage that, owing to the diverse results, different broad themes were incongruent for simultaneous analysis. On the basis of this decision, the data corpus was divided into 4 separate data sets (Figure 1). The data presented in this study were themes falling under the “information-seeking” data set (represented as a black line) that WB and RD independently identified. Once this new data set was determined, WB and RD performed data immersion, familiarization, and refinement of themes under the “information-seeking” data set. We reviewed and renamed themes before the final themes were determined. Notably, the gray line themes identified are not discussed in this study.

## Results

### Participant Characteristics

A total of 54 participants were contacted and invited to participate in a semistructured interview. A total of 22 (79%) of the 28 participants in the IC arm and 16 (62%) of the 26 participants in the SCC arm agreed to be interviewed. Participants were predominantly from the United Kingdom, the United States, and Australia or New Zealand. The mean age of the participants in the SCC and IC was 50.7 (SD 15.6) years and 48.1 (SD 12.3) years, respectively, and almost 89% (34/38) of the participants were female (Table 1). Participant characteristics were similar across the IC and SCC; however, there was a higher proportion of people with relapsing-remitting MS and men from the IC compared with the SCC.

**Table 1:** Participant sociodemographic and clinical characteristics (n=38).

Characteristics		SCC^a^ (n=16)	IC^b^ (n=22)
**Sex, n (%)**			
	Male	1 (6)	3 (14)
	Female	15 (94)	19 (86)
**Country of residence, n (%)**			
	Australia or NZ^c^	2 (13)	5 (23)
	United States or Canada	5 (31)	5 (23)
	United Kingdom	4 (25)	3 (14)
	Other	5 (31)	9 (41)
**MS^d^ phenotype, n (%)**			
	RRMS^e^	9 (56)	18 (82)
	PPMS^f^ or SPMS^g^	4 (25)	3 (14)
	Other	3 (19)	1 (55)
Age (y), mean (SD)		50.7 (15.6)	48.1 (12.2)
MS duration from diagnosis (y), mean (SD)		6.9 (5.7)	5.4 (4.3)

^a^SCC: standard care course.

^b^IC: intervention course.

^c^NZ: New Zealand.

^d^MS: multiple sclerosis.

^e^RRMS: relapsing-remitting multiple sclerosis.

^f^PPMS: primary progressive multiple sclerosis.

^g^SPMS: secondary progressive multiple sclerosis.

### Themes

#### Overview

We identified 2 themes from the data: (1) “motivations for learning” described the motivations behind why participants seek web-based information in general and their reasons for engaging with the MSOC specifically, and (2) “MS information on the web” explored the relationships between participants and the web-based health information with which they engage.

Verbatim quotes are used to illustrate the themes. The themes identified from the “information-seeking” data set were similar across both study arms. Therefore, both study arms are reported together; however, example quotations from each study arm are provided where possible. The quotes are followed by the study arm (eg, IC or SCC) and participant number.

#### Motivations for Learning

The theme “motivations for learning” explored participants’ reasons for enrolling in and engaging with the MSOC specifically and why they sought MS-related information on the web in general. Furthermore, the impact of the diagnosis on the information-seeking behavior of participants was investigated, particularly relating to how these behaviors informed the desire of people living with MS for content related to self-management.

#### Effect of Diagnosis on Information Seeking

The participants described receiving an MS diagnosis as a critical event in their lives, which precipitated information-seeking behavior for health-related knowledge. The participants described difficulties in accessing timely, comprehensible, and relevant information at diagnosis, and the MSOC was considered positive compared with self-managed research. Furthermore, the participants mentioned gaining information as quickly as possible following diagnosis while filtering out unnecessary information from the “information overload” as follows:

I feel like [the online course] would’ve been perfect when I was first diagnosed. When I was just desperately searching for anything, for any information about [MS].

SCC7

The initial impact of the diagnosis resulted in a desire to learn as much as possible as fast as possible, and the participants reported readily accessing many different web-based resources:

I was newly diagnosed and was in a phase where I was trying to learn as much as I could about MS as fast as I possibly can.

IC4

I completely stopped everything in my life and turned my life in a completely different direction with my diagnosis. I jumped onto all kinds of bandwagons, let’s just put it that way.

SCC10

The impact of the diagnosis affected some people’s ability to process the volume and type of information available, leading to a need for the controlled delivery of information that could be taken at their own pace:

When you first get MS, you do get some information and it’s sort of just thrown at you.

IC2

When I was first diagnosed, it wasn’t that it was confrontational, it was just that it was too much.

SCC4

In addition to the information provided at diagnosis, some participants were advised by their HCPs to seek further information via the internet. For some participants, the timing of their diagnosis was relevant. For those who received their diagnosis during the pandemic, there was little alternative than to seek web-based information. In these instances, HCPs recommended reputable websites for information seeking:

When I was first diagnosed I was told “look, you know that there’s lots of misinformation out there and there’s lots of anecdotal experiential information out there.” [My neurologist] told me to stick to the recommended sources, the MS Trust and the MS Society. And so I did read all that and they have some good information and they run some good webinars.

IC19

I was diagnosed during lockdown and the advice given to me was, “you’re just gonna have to go look up stuff on the internet.” They told me to go to certain sites: MS Trust, MS Society. They’re the big ones in the UK.

SCC16

For some participants, the avoidance of information appeared to be functional within the early stages of adjusting to an MS diagnosis:

There was avoidance at first, probably denial, you know, the grieving stages. But that did not last long cause then I had to decide what medicine I was going to go on. So then I just read everything.

IC22

Many participants were selective in the information they sought following their diagnosis, with some preferentially seeking realistic and positively framed information. Some participants saw the MSOC as a source of positively presented information as follows:

When you get diagnosed, there can be a lot of negative information and obviously just in your own life, you can go through a lot of hard changes after being diagnosed. But the course was something that was really positive.

SCC6

#### Knowledge Acquisition for Self-Management

The participants sought knowledge to better understand their MS, with information on lifestyle modifications acting as a potential tool to facilitate self-management skills. For most participants, discovering novel information was the primary motivator for engaging with the web-based courses, and they were open to new ideas:

I wanted to hear something new since - this disease - everybody has an opinion on it.

IC17

I wanted to just learn more about this disease...Is there anything new that I can learn from it?

SCC8

Many participants reported that discovering new and specific information that was not available in previously explored web-based spaces was a principal reason for engaging with the MSOC, that is, participants felt that learning certain information about lifestyle behaviors would help them manage their MS and potentially improve future health outcomes:

Most of the information was available through online publications...in terms of literature, a lot of information is available already. So, I was after the in-between sort of information, things like amounts, for example, how much to eat, in terms of quantity, and in terms of what not to do.

IC5

For some participants in the SCC, the level of lifestyle-related information was not as complex or detailed as they had hoped:

I felt like some of the information was reinforcing information that I had already heard and sometimes I was hoping that maybe it went a little more in depth.

SCC7

Furthermore, this knowledge had to be easily comprehensible, especially for those with cognitive impairment, where the accessibility of information within the MSOC enhanced knowledge acquisition:

I think what the course really did for me was it underpinned what I had read. For me because I have cognitive issues, I need to really simplify things.

IC19

Moreover, the participants commonly reported that the importance of undertaking the course was to consolidate their existing knowledge base as opposed to learning new information. Others used the course to “fill in the gaps,” having previously undertaken their own research:

I thought that it’s very positive reinforcement because I’ve done my research, I am doing this course and it’s telling me the same things.

SCC4

#### Sourcing Information to Gain Control

The subtheme “sourcing information to gain control” focused on the individuals’ intentions to seek information, exemplified by their enrollment into the MSOC with the objective of enhancing their perceived control over their MS. The participants expressed the belief that acquiring a deeper understanding of MS and lifestyle modifications could substantially enhance their capacity to effectively manage and exert control over the disease:

I’m just trying to cover all bases. Trying to take some control of a condition that I don’t really have much control of.

IC19

I think I was just looking for a bit more information so I felt more informed...just so I felt more in control.

SCC16

The participants had a common sentiment of not wanting to be passive in their experience of living with MS:

Since I got diagnosed with MS I’ve learned a lot and I keep learning. I don’t wanna be one of those people that just sits back and does nothing, just accepts that the doctors said I got this horrible disease and then don’t look any further.

IC9

The knowledge gained led to an enhanced sense of capacity to manage MS:

[MS is] the disease without a cure and you’ve got no control over it. So it [knowledge] gives you that sense of some control at least.

SCC9

#### Finding Community for Support and Information

Some participants experienced isolation and a lack of face-to-face communication and other support. Some participants with limited resources or a smaller population in their country of origin sought web-based connections or engagement. The forum enabled a certain degree of interaction and support:

Because I don’t know other people in Taiwan with MS, I could see other people on the internet and I could relate to them, and they could be someone like me and I could see they were doing something for their health.

SCC3

Cause I was alone. I have nobody to talk to about my diagnosis. I didn’t know anyone with it...It was awful. I didn’t find any groups on the internet or on Facebook, and there was no Instagram at that time. I was kind of isolated.

SCC8

The participants felt supported by hearing others’ experiences of MS on the community forum:

I wanted to explore the learning experiences of other people with MS.

IC20

A bit of refreshing my understanding on what MS is and then also a bit more moral support.

SCC5

Some participants described feeling comforted knowing that others were participating even if they did not communicate with them:

I commented maybe once or twice in the forum. It was mostly seeing that there’s other people doing the course...

IC12

### MS Information on the Web

#### Overview

The theme “MS information on the web” encompassed a range of subthemes that explored how and where people living with MS accessed and engaged with web-based information and the value they attributed to different spaces and their unique roles. People living with MS seek information from a variety of sources on the web; however, not all sources are considered equal in quality. Furthermore, this theme explored challenges associated with accessing MS-related information on the web, including conflicting opinions on disease management approaches and negative presentations of MS. Finally, the role that HCPs play in facilitating or supporting web-based information dispersal is discussed.

#### Where People Living With MS Find Information

The most commonly reported source of frequently accessed information on the web was via Facebook groups. Many participants used Facebook to gather more information about MS and understand others’ experiences of living with MS:

I joined all these MS groups on Facebook too because I’m just hoping to get the science.

IC4

Facebook groups, even in Ukraine, are where participants discuss how they change their life. They share their knowledge. They share their experience of different diets, Wahls and the others.

SCC8

In addition, Facebook was a useful tool for people living with MS to connect to others where no other meeting place existed:

There is a group on Facebook that is called “live with MS”...And maybe a few years after I was diagnosed, I was thinking about it, cause I was alone.

SCC8

I facilitate a Facebook group for Atlantic Canada because we’re not as big as Melbourne. We have a number of Atlantic Canadian provinces in one group. And because of the pandemic, things have gone mostly online, especially with the MS crowd.

SCC4

MS society websites were, in general, described as being useful, particularly at the point of diagnosis. In addition, MS societies provided an initial point of contact for people living with MS to access web-based information, where advice on where to seek further information was available.

Some participants used the Overcoming MS charity website and the related Overcoming MS Facebook group with mixed opinions:

I’ve been on the [OMS] website and MS Facebook groups... where there’s so much support as well... The OMS Facebook group, that would be most likely where they would get similar support. Even though it is not always a professor that answers; it’s really peer support.

IC16

I was also on the OMS support group on Facebook... actually I got off the Facebook group in the end. I thought “it’s not doing anything for me.”

IC11

The Overcoming MS website was cited by some participants in both the intervention and standard care arms as a useful source of information, particularly as it related to mindfulness or meditation:

Often I’ll do 15 minutes of just sitting and meditating in the morning and in the evening. And then when I lay down in the middle of the day, if I do, then I’ll do a mindfulness thing off the OMS website.

IC11

I’ve done some OMS webinars. And again, they haven’t been fully as informative as I would like but I am still doing them.

SCC10

#### Appraising Web-Based Information Sources

The participants in the MSOC consistently described the value of scientific evidence and trust in the sources of information when considering the nature of the web-based information they engaged with. Generally, participants appeared to have a high standard for evidence, and they were vigilant of unsubstantiated information, relying mainly on the reputation of the web-based source to formulate discretionary approaches to seeking web-based information:

I don’t believe anything which isn’t scientifically proven. That’s just my analytical mind.

IC8

I really need information that is not “somebody said something somewhere,” but some scientifically proven information; checked information.

SCC8

The method through which the participants critically appraised sources of information was primarily through name recognition and referrals from trusted institutions.

You guys were in my mind “you’re a reputable institution doing meaningful work”, and I had heard about the study through like that society and through my neuroscience center.

SCC7

I saw the Melbourne University so I thought it’s like “alright, I’ll do that.”

IC8

Similarly, the participants were especially conscious of misinformation on the web, whether from perceived experts or from other people living with MS:

There’s a lot of hoax things on the internet on how to cure, how to manage, how to do this and that.

IC8

I’ve joined a few sort of forum type things to find a lot of information from there. I tend to apply a sort of 80 / 20 rule to a lot of these things. It’s like 20% of it’s probably rubbish and 80% is okay.

SCC16

I have looked up things in Google, which has brought me to a blog or something, and I don’t care for blogs. I think a lot of the information is negative and the only things I really investigate on my own are the studies or research.

SC11

#### Selective Information Seeking

In addition to appraising web-based information sources, many people living with MS were discriminatory in the content of MS-related information sought on the web, choosing to avoid web-based information or describing a hesitancy toward information seeking, whereas others were keen to learn as much about MS as they could. Notably, many participants described a shift from avoiding information early in their disease to actively seeking it later with time:

I decided I don’t want to read anything about anyone’s progress getting worse. That’s why I was seeking out positive stories.

IC12

Conversely, many people living with MS were not particularly concerned with encountering negatively valanced information on the web:

I’ve wanted to get as much information, knowledge about my MS as I can. I’m one of those people.

IC9

There are a lot of things that will hit you like a ton of bricks whether you try and avoid it or not...

SCC6

## Discussion

### Principal Findings

People living with MS frequently seek MS-related education information, with the knowledge acquired playing an important role in facilitating self-management of the disease [2,3]. Using thematic analysis to understand how a cohort of people living with MS seek and engage with information related to MS across web-based platforms, this study provides an increased understanding of the information sources and needs of the people living with MS. Drawing from a larger data corpus within the context of the MSOC RCT, we identified how people living with MS seek MS-related information and the perceived barriers they face in accessing and integrating information. Two overarching themes emerged from the data: (1) “motivations for learning,” which explored individuals’ reasons for seeking and engaging with web-based information in general and the MSOC course specifically and (2) “MS information on the web,” pertaining to where the people living with MS seek web-based information and their experiences of sourcing this information.

### Comparison With Prior Work

#### Motivations for Learning

Understanding an individual’s motivation to access the MSOC and other web-based information is crucial for identifying personal attributes that may precipitate information seeking and influence both the sources and content of web-based learning. Although there has been a substantial qualitative investigation into the informational needs of people living with MS at diagnosis [10,28-34], including a meta-synthesis of qualitative studies looking at diagnosis more broadly [35], the personal behavior and experiences of people living with MS seeking web-based information, especially at diagnosis, have previously been underinvestigated. Notably, this study identified the time of diagnosis as a crucial time point that precipitated information seeking.

Despite concerns around the disclosure of an MS diagnosis [36], respondents described being encouraged to seek web-based information by their HCPs in addition to being independently motivated to seek information. Subsequently, people living with MS were often overloaded by a great deal of information, which at times led to confusion and jumping “on all sorts of bandwagons.” The participants in this study considered this initial self-guided “informational rush” as an intense experience and not necessarily always productive, possibly increasing the likelihood of engaging with misinformation, particularly for newly diagnosed people living with MS [13,31]. The negativity of information found at diagnosis was previously described in a similar study by Solari et al [29].

Although the experience of seeking information was confronting for many participants in the early phase of diagnosis, the process of information seeking itself appeared to provide support as individuals’ access to information may have facilitated a sense of illness coherence (ie, understanding of their MS). This is congruent with prior quantitative findings suggesting that a greater sense of illness coherence may assist people living with MS in managing their emotions [37], which in turn may facilitate seeking MS-related knowledge in a more measured manner that may result in people living with MS accessing more relevant knowledge. Relatedly, many participants reported that participating in the MSOC was rewarding and identified the positive way in which information was presented as the reason. This presents an important consideration for designers of educational content targeted at people living with MS, whereby balancing negativity and accuracy continues to be critical for engaging people living with MS [38].

The importance of MS-related information seeking following diagnosis was further emphasized through respondents’ reports that information presented at diagnosis within the health care setting was often too much or too little. Consistent with previous reports [29,30,32], respondents were generally dissatisfied with the information coupled with an emphasis on self-directed information seeking and learning that they received at the point of diagnosis. The task of self-directed information seeking proved challenging for some participants, which may have been a result of the overwhelming amount of information on the web and having to discern its trustworthiness [6,9]. This emphasizes the importance of personalized care at the time of diagnosis during this confronting period [39], including supplying information to patients guided by their readiness and desire for such information. Furthermore, our findings indicate that providing guidance to people living with MS on what type of information to source on the web and where to source information is an important priority for HCPs, particularly around the time of diagnosis. This may be of particular relevance in the current climate, with respondents reporting greater reliance on web-based sources at diagnosis following the COVID-19 pandemic, which has previously been described as less desirable by people living with MS [29].

The desire for enhanced capability in self-managing MS served as an important motivator for information seeking. In the process of simply seeking and gaining new knowledge, the participants hoped to have a sense of greater capacity to manage their MS. Personal control perceptions, referring to an individual’s sense of empowerment regarding the effectiveness of personal coping behaviors [39], have been reliably linked to motivation for self-management in chronic disease populations [40], lower levels of depression in people living with MS [41], and better quality of life. Information seeking for this purpose was perceived as a protective action compared with a passive or fatalistic approach to disease management.

#### MS Information on the Web

The second major theme identified was the sourcing of MS-related health information on the web by the study participants and their relationships with these sources. Facebook was the most commonly reported source of web-based information through official institutional MS Facebook pages or Facebook community groups. Facebook provided not only direct educational content for people but also a sense of peer support where asynchronous communication could take place. Furthermore, Facebook was useful for creating new communities in areas where they previously did not exist, particularly in countries with smaller populations or a low prevalence of MS.

Seeking information through communication with other patients is a common theme identified among chronic disease populations [42], and it represents a motivating factor for people to continue to engage with or develop these web-based communities [43,44]. Furthermore, information seeking through MS Society Facebook pages or charitable organizations such as MS Societies and the Overcoming MS website was also common among the participants. MS Society websites were recognized as an initial point for health information, particularly around the time of diagnosis, whereas the Overcoming MS website was often sought by people living with MS seeking information specifically related to lifestyle modification. Overall, our findings suggest that people living with MS engage with the Facebook community, organization groups, or MS Society websites for general information seeking, information clarification, and community support. Through these web-based sources, people living with MS are able to search for filtered information according to their health needs, such as lifestyle modification information.

As reported previously, the role of trust was of great importance to participants when encountering information on the web [45]; it was emphasized that the respondents required the information presented to be scientifically validated. Although some respondents reported having some scientific skills (eg, identifying studies and interpreting research findings), which facilitated the critical appraisal of information on the web, most respondents relied on the name recognition of institutions. Similar to other studies [6,9,10], misinformation was still a considerable concern for respondents who were cautious of material from unfamiliar sources, particularly information presented as testimony or hearsay from others. Building on the results from prior studies, this study adds context to challenging web-based misinformation, whereby trust in institutions can play a key role in engaging people living with MS with appropriate health content.

People living with MS described encountering negative health-related information on the web frequently. There were 2 common responses to encountering negative information: avoiding or accepting. The most common reason for avoiding information was seeing portrayals of disability. Other studies have similarly described how people living with MS struggle with encountering depictions of disability on the web, particularly close to the diagnosis [28,38]. According to some participants, the immediacy of addressing management options and health concerns played a role in overcoming their initial avoidance of confronting information and ushered them toward broad information seeking. Another subset of participants found it best to accept the presence of negative information from the outset, as this was impossible to avoid when seeking MS-related health information.

### Strengths and Limitations

This study has strengths in consolidating the common reasons for seeking web-based MS-related information and preferences for information types and capturing individuals’ experiences with this information. However, this study has some limitations. First, this qualitative study population was drawn from a larger RCT in which people living with MS self-selected their involvement with the study through voluntary participation. This may limit the generalizability to the broader MS population as participants who seek and choose to enroll in web-based programs for MS may possess higher levels of patient activation compared with the general population. Furthermore, the participants completing the course, baseline study, and postevaluation survey and consenting to postcourse interviewing may represent an even more select and motivated subpopulation. Second, the views expressed are those of the interviewed participants and do not represent all potential views. Some participant characteristics are underrepresented in this cohort, such as the number of men and individuals with more progressive MS phenotypes, which may further limit generalizability.

### Conclusions

This study of a subset of participants in the MSOC RCT explored information seeking and reasons for seeking this information and engaging with the MSOC and other web-based information. Notably, this study identified previously underexamined barriers to people living with MS seeking information, including the timing of diagnosis and the validity of information available on the web. These factors have the potential to influence individuals’ experiences of engaging with health information on the web. These findings should assist HCPs, researchers, and policy makers in determining optimal ways to provide reliable educational support to people living with MS, especially at critical points such as MS diagnosis. Ultimately, as people living with MS continue to engage with increasingly prevalent web-based resources related to their health, a greater understanding of the information they seek is critical for ensuring that they gain the maximum benefit of information in terms of content and potential impact.

## Appendix

Multimedia Appendix 1 Interview schedule of questions for participants.

## Abbreviations

ACTRN: Australian and New Zealand Clinical Trials Registry
HCP: health care professional
IC: intervention course
MS: multiple sclerosis
MSOC: Multiple Sclerosis Online Course
RCT: randomized controlled trial
RTA: reflexive thematic analysis
SCC: standard care course

## Data Availability

The data sets used for quantitative analysis and anonymized transcripts used for qualitative analysis are available from the corresponding author upon reasonable request.
