# Supplementary material for: Web-Based Health Information Seeking by People Living With Multiple Sclerosis: Qualitative Investigation of the Multiple Sclerosis Online Course
Source: J Med Internet Res. 2024 Feb 9;26:e53372. doi: 10.2196/53372 (PMC10891496; doi:10.2196/53372)
Supplement: Multimedia Appendix 1 [file jmir_v26i1e53372_app1.docx]

**An online multiple sclerosis educational course RCT: a qualitative analysis**

**Post-course interview Schedule**

As part of the MSOC effectiveness RCT we propose two qualitative interviews of a randomly selected group of participants from both the standard-care course and the intervention course

**Setting:**

Thank you for speaking with me.

I am ….., a researcher from the Neuroepidemiology Unit at the University of Melbourne.

You have recently taken part in the first University of Melbourne’s MS Online Course. We are speaking with you today to understand your experiences of the course, what worked well for you and what didn’t, so we will have a better understanding of the experience from participants’ perspectives. The interview should take 30-40 minutes. The information we gather today will be transcribed in to a written anonymous form. We hope to use the information we gather from this interview to inform the future development of the course and to potentially publish our findings.

May I confirm that you consent to participate in this interview and for us to analyse and potentially publish the findings from this study.

Do you have any questions before we begin?

Thank you. Are you ready to begin?

**One month post course completion. Prompts are in light grey**

Aims: To assess:

- 1. Participants’ views regarding motivation to undertake the course
  2. Views of the content of the course
  3. Participants’ experiences
  4. Use of community
  5. Initial changes to lifestyle
  6. Initial changes to attitudes to MS, health and the future

1. What was your motivation for enrolling in this research project?
   1. Was there anything about the research aspect of the course?
   2. Was it mainly for your own reasons…what were they?
2. Was there anything you hoped to gain or experience from undertaking the course?
   1. Were you specifically interested in lifestyle modification?
   2. Were you interested in evidence about MS and lifestyle?
   3. Were you interested in being part of a community?
   4. Were you looking for something else: control, confidence, confirmation?
3. What was the level of information provided like for you?
   1. Was there new information?
   2. Was there information you already knew?
   3. Was it pitched at the right level for you?
   4. What would you like more of? More advanced information about the modules? More links to articles?
4. How did you find the community forums?
   1. Did you engage with the forum?
   2. What things encouraged or prevented you from using the forum?
   3. Did you engage with other people with MS? What comments do you have about that engagement?
   4. Did you engage with the facilitators? What comments do you have? the facilitators?
   5. What could make it better?
   6. Did you engage in the final ceremony?
   7. Was the engagement of any of importance to you?
5. Is there anything about your lifestyle you have changed since taking the course?
   1. If so, what have you changed? Prompt, your daily routine to your exercise, diet, meditation, Vit D, Omega 3
   2. Why didn’t you make any changes?
6. Have your attitudes about your MS and your future changed
   1. Control
   2. Confidence
   3. Uncertainty
   4. Lifestyle changes
   5. Motivation
   6. View of the future
7. Is there anything else you would like to say about the course?

Do you have any questions?

If we have raised any concerns for you please feel free to contact us via the contact details on the email that was sent to you. We can also give you details of external agencies to assist if needed.

1. For Australia: HeadSpace <http://headspace.org.au> or Lifeline on 131114 in Australia.
2. For Canadian: Toronto Distress Centre at either <https://www.torontodistresscentre.com/> or via phone 416-408-HELP (4357) at any time of day in Canada.
3. For NZ: Lifeline at 0800 543 354 (0800 LIFELINE) or free text 4357 (HELP) or visit the website <https://www.lifeline.org.nz/>.
4. For UK: Samaritans via phone at [116 123 (UK)](https://www.samaritans.org/how-we-can-help-you/samaritans-free-call-helpline-number-faqs) or [116 123 (ROI)](https://www.samaritans.org/how-we-can-help-you/contact-us/calling-samaritans-roi) 24/7 or by visiting <https://www.samaritans.org/> .
5. For US: The participant will be recommended to consult with Suicide Prevention and Crisis Hotline 1800-273-8255 at any time in the United States.
